# Evolutionary history of *Daphnia* drives divergence in grazing selectivity and alters temporal community dynamics of producers

**DOI:** 10.1002/ece3.3678

**Published:** 2017-12-10

**Authors:** John S. Park, David M. Post

**Affiliations:** ^1^ Committee on Evolutionary Biology University of Chicago Chicago IL USA; ^2^ Ecology & Evolutionary Biology Yale University New Haven CT USA

**Keywords:** consumer dynamics, life histories, multitrophic, primary producers, temporal composition

## Abstract

Consumers with different seasonal life histories encounter different communities of producers during specific seasonal phases. If consumers evolve to prefer the producers that they encounter, then consumers may reciprocally influence the temporal composition of producer communities. Here, we study the keystone consumer *Daphnia ambigua,* whose seasonal life history has diverged due to intraspecific predator divergence across lakes of New England. We ask whether grazing preferences of *Daphnia* have diverged also and test whether any grazing differences influence temporal composition patterns of producers. We reared clonal populations of *Daphnia* from natural populations representing the two diverged life history types for multiple generations. We conducted short‐term (24 hr) and long‐term (27 days) grazing experiments in equal polycultures consisting of three diatom and two green algae species, treated with no consumer, *Daphnia* from lakes with anadromous alewife, or from lakes with landlocked alewife. After 24 hr, life history and grazing preference divergence in *Daphnia ambigua* drove significant differences in producer composition. However, those differences disappeared at the end of the 27‐day experiment. Our results illustrate that, despite potentially more complex long‐term dynamics, a multitrophic cascade of evolutionary divergence from a predator can influence temporal community dynamics at the producer level.

## 1 INTRODUCTION

The life history of consumers in seasonal ecosystems significantly influences the ecology of primary producers (Carpenter, Fisher, Grimm, & Kitchell, 1992; Thackeray et al., 2010; Winder & Schindler, 2004). For example, variation in the timing of emergence or migrations and population growth rates of consumers can alter the intensity and timing of grazing, which can in turn shape producer production and community composition through time (West and Post, 2016; Winder & Schindler, 2004; Metzger, Coughenour, Reich, & Boone, 2005). Variation in the temporal nature of consumption is often linked to interannual variation or variation among species, but it can also emerge from divergence among populations. This intraspecific variation in the life history of consumers may also be of considerable importance to community structure and dynamics (Bolnick et al., 2002; Post, Palkovacs, Schielke, & Dodson, 2008; Walsh, Pierre, & Post, 2014). Developing a better understanding of how consumer life history evolution and the resulting variation influences communities is critically important for studying food web ecology and seasonal ecosystem function, especially in the face of a rapidly changing climate associated with decoupled multitrophic interactions.

Here we ask whether the intraspecific life history divergence of a keystone consumer, *Daphnia*, might influence the temporal patterns of producer community composition. *Daphnia* are the dominant grazers in most lakes and important for both energy flow to upper trophic levels and trophic cascades (Pace, 1984; Carpenter et al., 1987; Lampert 2011). As the dominant grazer, life history variation among and within species of *Daphnia*, particularly related to population growth rates, may have strong impacts on algal community structure and dynamics through time. The timing and duration of when a species is present is not only a function of the abiotic variables of the habitat, but also of its interactions with other species within and across trophic levels at various points through seasons (Elzinga et al., 2007; Korhonen, Soininen, & Hillebrand, 2010; Matthews & Pomati, 2012). Divergence in the pattern of population growth and grazing can thus affect the interplay between consumer and producers through the annual cycle and influence the phenological patterns within producer communities. On the other side of the same coin, significant shifts in producer temporal turnovers can also have feedback effects on consumer populations which also have particular annual phenological schedules. Thus, the timing of food availability and diversity can have bottom‐up effects on consumers and predators and ultimately impact the entire food web.

**Figure 1:**
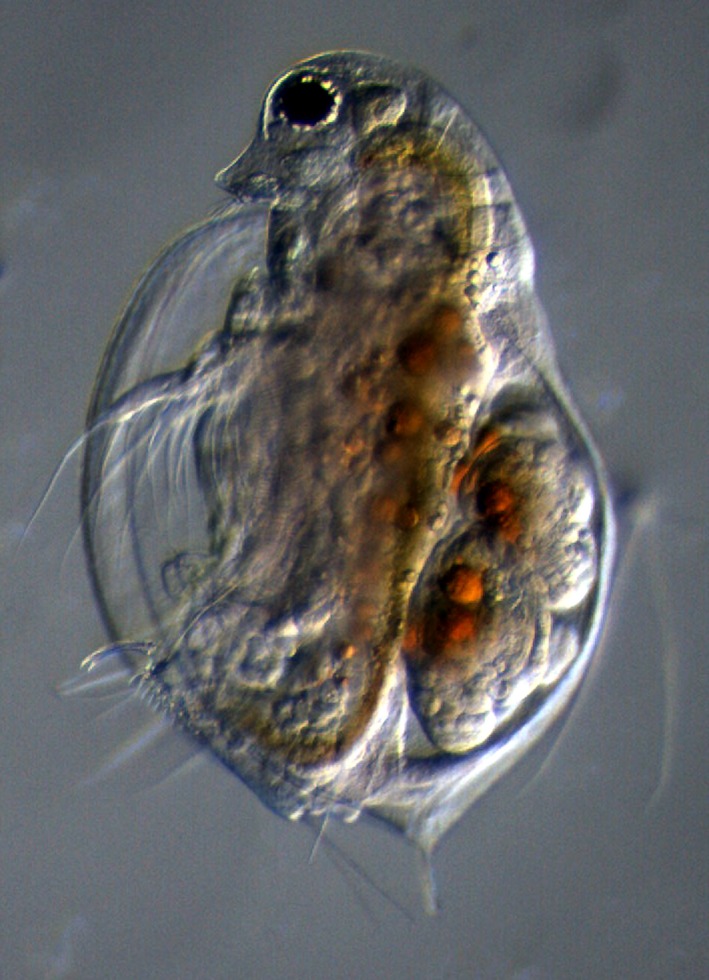
Grazer zooplankton *Daphnia ambigua* (photograph by DM Post)

We take a multitrophic system involving a model grazer zooplankton *Daphnia ambigua* (Figure 1) and a community of phytoplankton species from New England freshwater lakes to address the impact of consumer life history divergence on composition change in producer communities. Post et al. (2008) and Walsh and Post (2011) demonstrated that intraspecific life history divergence in the freshwater predatory fish alewife (*Alosa pseudoharengus)* has selectively driven a divergence in the life histories of *D. ambigua* in New England lakes. In lakes with anadromous alewife which migrate annually between freshwater and the Atlantic Ocean, *D. ambigua* have a higher juvenile growth rate, mature earlier, produce larger offspring clutches, and, as a consequence, have higher population growth rate (Walsh & Post, 2011). *D. ambigua* from lakes with anadromous alewife (hereafter “anadromous lakes”) also invest more in sexual reproduction and have a greater plastic response to the presence of alewife than *D. ambigua* from lakes with landlocked alewife (hereafter “landlocked lakes”) that spend their entire lives in freshwater (Walsh & Post, 2012). The combination of different seasonal patterns of predation by alewife and life history differences allows *D. ambigua* in anadromous lakes to reach very high densities in the spring, potentially inducing different seasonal patterns of grazing in phytoplankton communities than *D. ambigua* in landlocked lakes that are present in the water column at low densities throughout the year (Post et al., 2008).

Phytoplankton communities also exhibit significant and predictable patterns over a year in lakes. For example, species of the green algae group *Chlorophyta* are about 15 times more abundant than cyanobacteria between April and May, but cyanobacteria become about 5 times more abundant than *Chlorophyta* between July and August (Walsh et al., 2014). Therefore, as *D. ambigua* in lakes with anadromous alewives are present only during the spring, they do not experience the high abundances of cyanobacteria of summer that *D. ambigua* in landlocked lakes do. As such, populations of *D. ambigua* of divergent life histories in these seasonal lakes co‐occur with different phytoplankton assemblages.

Daphnia are generalist grazers that filter a wide variety of phytoplankton, but have the ability to discriminate among phytoplankton and influence phytoplankton species composition (Porter, 1977; DeMott,^1982^; Epp, 1996; Sarnelle, 2005). Phytoplankton species composition can influence grazing performance and population growth rates in *Daphnia* (Narwani and Mazumder, 2010; Walsh et al., 2014) and *Daphnia* can evolve rapidly in response to variation in phytoplankton abundance and composition (Hairston et al., 1999). Here, we address a related question by asking how life history evolution in Daphnia might influence the grazing effect of Daphnia on phytoplankton composition. We hypothesize that life histories that diverged *in situ* in anadromous or landlocked lakes, which govern their temporal presence and population growth rates of *D. ambigua*, are associated with differences in preference and intensity of grazing on phytoplankton species, potentially shifting community structure. Previous studies have shown that the life history evolution of *D. ambigua* in anadromous and landlocked lakes has a large impact on the dynamics of a single, highly edible species of phytoplankton (*Scenedesmus*; Walsh & Post, 2011; Walsh, DeLong, Hanley, & Post, 2012). Here we ask whether this life history evolution can influence algal community composition when *D. ambigua* are provided a mixed species community. We evenly mix five broadly representative genera of phytoplankton, including green algae and diatoms, and ask the following: 1) If intraspecific *Daphnia* life history divergence has driven differences in grazing selectivity and 2) if selectivity divergence influences the phytoplankton community composition over multiple generations.

## 2 METHODS

We examined the influence of evolutionary history of *Daphnia ambigua* on the temporal dynamics in phytoplankton community structure. Evolutionary history was divided into anadromous and landlocked lake types (with respect to the life history of the predator, alewife), which has driven significant life history divergence in *Daphnia*. We hypothesized that differences in grazing selectivity could exist between *Daphnia* associated with anadromous and landlocked alewife life histories, and that this difference may impact the way phytoplankton communities change through time differently. For our experiments, we collected *Daphnia from three lakes in southern New England with anadromous alewife*(“Bride,” “Dodge,” and “Gorton”) and three lakes with landlocked alewife (“Amos,” “Long,” and “Quonnipaug”).

### 2.1 Daphnia ambigua ***Rearing***

We extracted ephippia (chitin‐encased eggs in diapause) of *Daphnia ambigua* from sediment collected from the six source lakes. Sediment was collected from each lake using an Ekman grab at the surface of the benthos, in order to sample ephippia representative of contemporary genotypes. We hatched ephippia in well plates in *Daphnia*‐COMBO solution (Kilham et al., 1998) and obtained eight genetic clones from each lake. As soon as juvenile *Daphnia* hatched, we transferred these first laboratory generation individuals from each clone into 50 ml beakers (only one clone per beaker) with COMBO and an abundant supply of the chlorophyte *Chlamydomonas moewusii* (concentration: >1.0 mg C L^−1 ^day^−1^) as a common food source. The typically used *Daphnia* food *Scenedesmus obliquus* (as in Walsh et al., 2012) was not used during rearing, as it is competitively dominant over many other phytoplankton taxa in laboratory mesocosm settings, and we were concerned about its invading and dominating in later experiments that would obscure grazing preference differences of *Daphnia*. We reared the *Daphnia* clones in an incubator with constant photoperiod (12L:12D cycle) at 17°C. For the second laboratory generation, we took three offspring from the first clutch in each beaker into a new beaker with fresh *Daphnia*‐COMBO and abundant *Chlamydomonas*. Thereafter, we propagated each clonal population by randomly dividing the population into new beakers as soon as offspring appeared (every ~7 days), providing fresh medium and algae every 3–4 days, to keep the density of *Daphnia* under 15 individuals per beaker at all times (as per Walsh et al., 2012). We maintained the same incubator conditions and rotated the placement of beakers in the incubator randomly every three days to minimize any bias from light and temperature distribution inside the incubator. With this process, we produced a stock of *Daphnia* individuals that consisted of eight distinct genetic clones from each of the six lakes (6 × 8 = 48 clones) which had experienced at least five generations of laboratory rearing in order to minimize maternal effects on grazing experiments.

### 2.2 Phytoplankton cultures

During the *Daphnia* rearing phase described above, we simultaneously maintained cultures of *Chlamydomonas* in Algae‐COMBO medium in 2L flasks with oxygen bubblers at room temperature and 12L:12D photoperiod, to provide food for the *Daphnia* clonal populations.

In preparation for the experiments described below, we employed the same culturing method for five different taxa of phytoplankton that would make up the common garden community. Cultures were directly isolated from Southern Connecticut lakes or purchased from the University of Texas at Austin Culture Collection (UTEX). These included three diatom species (Bacillariophyta)—*Asterionella sp*. (Pattagansett Lake, CT, USA)*, Cyclotella quillensis* (UTEX–LB FD142)*, and Navicula sp*. (Bride Lake, CT, USA)—and two Chlorophyceaen green algae species—*Mougeotia sp*. (Rogers Lake, CT, USA)*, Chlamydomonas moewusii* (UTEX–1053). These five taxa were chosen to be ideal for common garden experiments because 1) they are representative of the dominant growth forms of phytoplankton communities in Southern Connecticut lakes which the *Daphnia ambigua* populations consume in their natural habitats, 2) they represent a broad and relevant diversity of functional traits, including growth form and cell size, 3) none is known to be competitively dominant over others, and 4) they grow easily in laboratory cultures (Weis, 2016; Weis & Post, 2013).

### 2.3 Short‐term grazing experiment

In preparation for the short‐term grazing experiment, we isolated two adult individuals that had reproduced at least once from each of the eight clonal populations, from each lake. We then transferred the 16 individuals (2 individuals × 8 clones) from each lake into a single 50‐ml beaker with *Daphnia*‐COMBO but no algae. We repeated the above three times so that we obtained three replicates of mixed‐clone populations for each lake (2 lake source types × 3 lakes per type × 3 replicates = 18 beakers, with 16 individuals each). We starved these 288 *Daphnia* individuals for 24 hr in a dark incubator and serially transferred them individually through three autoclaved test tubes containing uncontaminated medium, to ensure the individuals were clear of any algal cells prior to the experiment.

For the short‐term grazing experiment, we transferred equal and known densities of each phytoplankton species into 250‐ml flasks. We equalized phytoplankton densities by 1) calculating the average of four cell number counts inside a 0.9‐mm^3^ hemocytometer grid for each of the five phytoplankton cultures, 2) then calculating the volume needed to obtain 8 × 10^6^ cells of each phytoplankton species, and 3) pipetting the calculated volumes from each culture into the flasks. The number 8 × 10^6^ was calculated after preliminary trials to reflect a density similar to *Daphnia* rearing conditions (~40,000 cells ml^−1^ species^−1^), as well as to create polyculture mesocosms with a volume of 200 ml.

We produced 21 such flasks, 18 with mixed‐clone *Daphnia* populations described above and three left *Daphnia*‐free as controls. We placed these flasks, covered, in a dark incubator for 24 hr to allow feeding and minimal phytoplankton growth. After the 24‐hr period, we calculated the average of four cell number counts for each phytoplankton species inside a 0.9 mm^3^ hemocytometer grid.

### 2.4 Long‐term dynamics experiment

Prior to the 27‐day long‐term dynamics experiment, we repeated the same preparation procedures as the short‐term grazing experiment; however, instead of 250‐ml flasks we used 8‐L fish tanks filled half way to create larger mesocosms, and started with much lower densities of each phytoplankton species compared to the short‐term experiment (~3,000 cells ml^−1^ species^−1^). We transferred the same clonal mixtures of *Daphnia* from each lake (two adult individuals per clone) and again replicated them three times and made three control mesocosms with no *Daphnia*. We placed the 21 tanks in an incubator in a 12L:12D photoperiod cycle at 17°C. Every three days for the duration of the experiment we 1) stirred each mesocosm thoroughly and randomly switched positions in the incubator and 2) sampled *Daphnia* population sizes in each tank by taking the average of two 80‐ml subsample counts of individuals, in order to determine whether any population size differences affected grazing patterns. We returned all subsampled individuals to the tanks immediately after measurement. At the end of the 27‐day period, we measured cell numbers of the phytoplankton species using the same methods as for the short‐term experiment.

### 2.5 Statistical analyses

We analyzed all data using R (RStudio, Version 3.2.2). In the middle of the long‐term experiment, *Daphnia* population in one of the nine landlocked lake treatment replicate tanks crashed on day 15, presumably due to an infection. This replicate was omitted from subsequent analyses. For each polyculture community, lake type (Anadromous, Landlocked) was entered as a fixed effect, and lake populations were nested within type to test whether between‐lake differences strongly drove variance regardless of lake type. Replicates of each lake treatment were entered as blocking (random) factor. With this nested design, we employed a multivariate analysis of variance on the five‐species polycultures to compare community composition differences with respect to cell counts after log‐transforming the data. While log‐transforming count data can be statistically problematic if datasets contain observations of zeroes, our dataset contained no zeroes. We verified multivariate normality using the package “MVN” in R. We applied post hoc ANOVA tests on individual phytoplankton species to further investigate structural difference in composition and determine the species most strongly driving the community composition differences.

## 3 Results

Evolutionary history of *Daphnia* significantly influenced phytoplankton community structure over a short term (24 hr), suggesting intraspecific divergence of grazing preference; however, differences in phytoplankton composition disappeared after a longer experiment (27 days).

### 3.1 Short‐term grazing experiment

**Figure 2:**
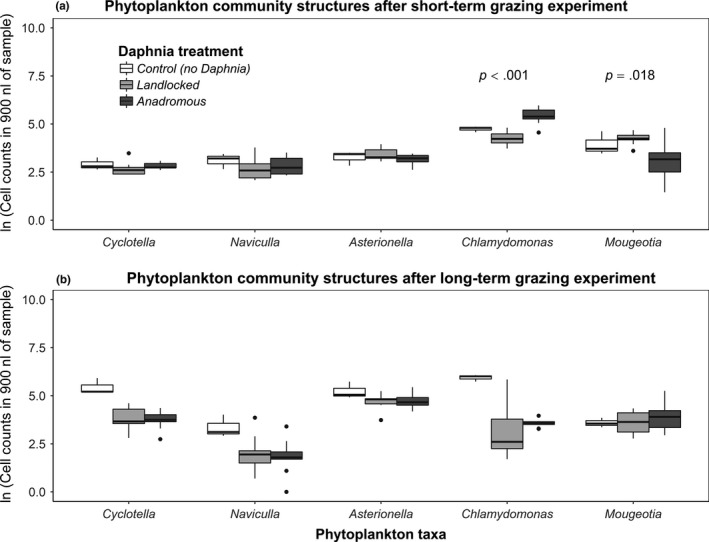
Phytoplankton community structures at the end of mesocosm experiments that began with equal densities of phytoplankton taxa, and treated with no *Daphnia* (control), *Daphnia* from three different landlocked alewife lakes, and *Daphnia* from three different anadromous alewife lakes. *p*‐values are indicated above phytoplankton taxa that were significantly different between landlocked and anadromous treatments (nested MANOVA, lake within lake type)

We observed significant phytoplankton community structure differences among polyculture mesocosms treated with *Daphnia* from anadromous, and *Daphnia* from landlocked lakes after 24 hr of feeding in the dark (Figure 2a). Data were multivariate normal (Mardia's multivariate skewness *p *=* *.616, kurtosis *p *=* *.386). With lake identity nested within lake type, there was a significant lake type effect (MANOVA, Pillai's statistic = 0.767, *df* = 1, *p = *.011) and a nonsignificant lake effect (MANOVA, Pillai's = 0.432, *df* = 5, *p = *.321). Our results suggest that evolutionary history of *Daphnia* induced a significant difference in phytoplankton community structure over the short‐term experiment via grazing selectivity. Post hoc nested‐ANOVA analyses showed *Chlamydomonas* to be the most significant driver of this community difference among lake types, followed by *Mougeotia*, and the other three were nonsignificant (Table 1).

**Table 1:** Post hoc ANOVA results for each species after the short‐term experiment, in common polyculture mesocosms treated with *Daphnia* from anadromous lakes or *Daphnia* from landlocked lakes. Source lake identity is nested within lake type, and analyses were performed on log‐transformed count data. Lake type *p‐*values <α = 0.05 are bolded

Species	*df* (lake type, lake)	*F*‐value (lake type, lake)	*p*‐value (lake type, lake)
*Chlamydomonas moewusii*	(1, 5)	(41.781, 2.131)	(<**.001**, 0.168)
*Mougeotia* sp.	(1, 5)	(7.369, 1.312)	(**.018**, 0.273)
*Cyclotella quillensis*	(1, 5)	(1.043, 0.659)	(.326, 0.431)
*Naviculla* sp.	(1, 5)	(0.045, 2.138)	(.835, 0.167)
*Asterionella* sp.	(1, 5)	(2.420, 0.055)	(.144, 0.817)

### 3.2 Long‐term dynamics experiment

After 27 days of ecological dynamics in tanks, differences in phytoplankton community structure (Figure 2b) treated with *Daphnia* from anadromous lakes, and *Daphnia* from landlocked lakes became nonsignificant (MANOVA, “lake type”: Pillai's = 0.106, *df* = 1, *p = *.948; “lake”: Pillai's = 0.545, *df* = 5, *p = *.150). Data were multivariate normal (Mardia's multivariate skewness *p *=* *.364, kurtosis *p *=* *.764).

**Figure 3:**
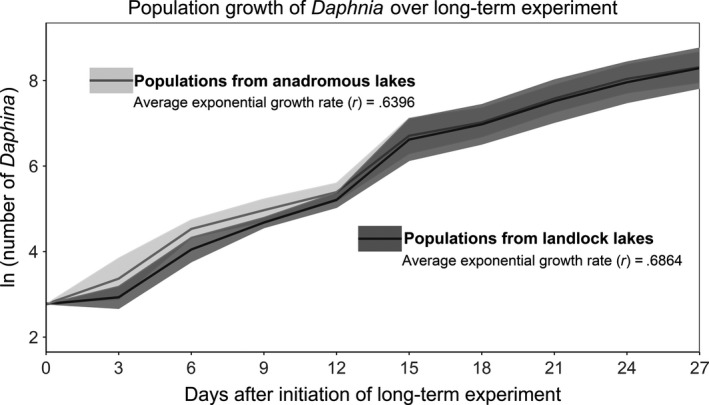
Growth of *Daphnia* populations in the long‐term 27‐day experiment, measured every 3 days. Each growth curve shows the average (± 1 SD) of replicate mesocosms comprising three lake sources of each type (landlocked and anadromous). Exponential growth rates (*r*) calculated from nontransformed data for each lake type are shown. Nested ANOVA indicated that the final population sizes of lake type treatments were not significant (*p *=* *.633)

The exponential population growth rates (*r*) in mesocosm tanks of *Daphnia* from anadromous (= 0.6396) and landlocked (= 0.6864) lake sources were similar over the course of 27 days (Figure 3). Population sizes of anadromous versus landlocked lake treatments at the end of the long‐term experiment were not statistically different (nested‐ANOVA, lake within lake type, *p = *.633). In previous studies where populations were reared in monocultures of the alga *Scenedesmus obliquus*,* Daphnia* from anadromous and landlocked lakes had significantly different growth rates (Walsh & Post, 2011; Walsh et al., 2012). Here, in polycultures consisting of five different phytoplankton species, we did not find significant differences in *Daphnia* population growth.

## 4 DISCUSSION

*Daphnia* populations evolve rapidly in response to environmental conditions including the abundance and composition of phytoplankton (Hairston et al., 1999) and seasonal patterns of predation (Walsh & Post, 2011). As the dominant grazer in lakes, evolution of grazing performance or population growth rates may strongly impact the temporal dynamics of phytoplankton community composition through two possible mechanisms. First, divergent *Daphnia* life histories may be associated with different per capita grazing rates, and thus, populations may exhibit different consumption patterns through time. However, Walsh et al. (2012) showed that *Daphnia* populations from anadromous or landlocked lakes did not exhibit a significant difference in per capita grazing rate. Second, *Daphnia* of divergent life histories may have evolved different preferences for the specific phytoplankton species they consume. Here we tested this second mechanism by introducing *Daphnia* of divergent life histories to common communities of phytoplankton species.

We found that *Daphnia* evolutionary history had significant effects on phytoplankton community composition over the course of 24 hr. The greatest difference in community composition was in the two green algae: *Chlamydomonas*, the only single‐celled, flagellated, and mobile species, and *Mougeotia*. Interestingly, *Chlamydomonas* abundance in anadromous *Daphnia* treatments was not only higher than that in landlocked *Daphnia* treatments, but it also rose above that in controls (with no *Daphnia*). We hypothesize two potential causes for this pattern. First, positive preference of anadromous lake *Daphnia* for the filamentous green alga *Mougeotia* (Figure 2a) may have caused a burst of nutrient release into the mesocosms via messy foraging (*sensu* Sterner, 1990; Urabe, 1993; Donk et al., 2010), which then accelerated population growth for the single‐celled and mobile *Chlamydomonas* over 24 hours. Another possibility is that *Chlamydomonas* in the anadromous *Daphnia* treatments may have had a strong compensatory response to greater grazer pressure (Bell, 2002; Steiner et al., 2011; Weis & Post, 2013), inducing an increase in population growth. But if this were the case, from our results it is yet unclear whether anadromous *Daphnia* had a stronger grazing preference for *Chlamydomonas* than did landlocked *Daphnia* and thus induced a stronger compensatory response, or if *Daphnia* from the two lake types induced an equal amount of compensatory response in *Chlamydomonas* but anadromous *Daphnia* had a lower clearing rate of *Chlamydomonas* than did landlocked *Daphnia*. No previous studies have investigated grazing rate differences between *Daphnia* of divergent life histories on phytoplankton species other than *Scenedesmus* in this system. Thus, it is unclear exactly where the consumption preference difference lies. However, our results show a strong grazing difference by *Daphnia* populations of divergent evolutionary histories, when introduced to a mixed community of phytoplankton species.

Despite a strong short‐term effect, divergence in phytoplankton community composition disappeared after 27 days. In the scope of our experiment, there are two potential explanations for the lack of a long‐term effect. First, frequency‐dependent consumption of phytoplankton may have dampened any proportionally faster growing taxa during the 27‐day period simply due to a rise in detection rate. Compounding that, life history and functional response differences among the phytoplankton species, in a mixed mesocosm, may have resulted in a complex intra‐guild maintenance of phytoplankton diversity. Second, if compensatory response of *Chlamydomonas* with anadromous *Daphnia* indeed drove initial differences seen in the short‐term experiment, it may have been a short‐term response only, which was not maintained through the long‐term experiment that lasted over multiple phytoplankton generations.

Our experiments began with uniform densities of phytoplankton species; but in lakes, phytoplankton species have different seasonal phenologies, and thus, their natural community composition is rarely, if ever, uniformly abundant through the nonwinter months. Lake populations of *Daphnia* have their own seasonal phenologies, which have diverged significantly depending on lake evolutionary history. Thus, *Daphnia* populations in the two types of lakes putatively encounter different communities of phytoplankton. In our common garden experiments, we simplified this natural orchestra of consumer and producer phenologies to ask whether grazing preference is different as a result of divergence in temporal occurrence of *Daphnia* in lakes. Our results suggest a strong divergence in preference.

Understanding intraspecific divergence of consumer preference is broadly relevant in two main ways. First, it provides the basis for a mechanistic understanding of phenological adaptation. With changing climates, the delicate balance of the phenology of seasonal organisms is shifting across various systems, aquatic, and terrestrial alike (Durant, Hjermann, Ottersen, & Stenseth, 2007). There is a large gap in our understanding of how organisms will biologically mitigate increased mismatches with the timing of resource availability (Winder & Schindler, 2004). Adaptive evolution of resource preference may be one mechanism. Secondly, a cascade of eco‐evolutionary change down trophic links can impact overall primary production and, ultimately, the functioning of ecosystems (Walsh et al., 2014). Our results support the growing consensus that life history evolution of predators and consumers can have community scale ecological effects (Bassar et al., 2012; Harmon et al., 2009; Post et al., 2008). Further, divergence in consumption pattern may influence not only just overall composition of resources and total primary production, but also the temporal succession of the composition and seasonal patterns of production.

## CONFLICT OF INTEREST

None declared.

## AUTHOR CONTRIBUTIONS

JSP conducted the experiments, analyzed the data, and wrote the manuscript. DMP contributed to writing the manuscript and provided partial funding.
